# Concentration-dependent protein loading of extracellular vesicles released by *Histoplasma capsulatum* after antibody treatment and its modulatory action upon macrophages

**DOI:** 10.1038/s41598-018-25665-5

**Published:** 2018-05-23

**Authors:** Ludmila Matos Baltazar, Daniel Zamith-Miranda, Meagan C. Burnet, Hyungwon Choi, Leonardo Nimrichter, Ernesto S. Nakayasu, Joshua D. Nosanchuk

**Affiliations:** 1 Department of Microbiology and Immunology, Albert Einstein College of Medicine, Bronx, New York USA; 2 Division of Infectious Diseases, Department of Medicine, Albert Einstein College of Medicine, Bronx, New York USA; 3 Biological Sciences Division, Pacific Northwest National Laboratory, Richland, Washington USA; 4 Saw Swee Hock School of Public Health, National University of Singapore, Singapore, Singapore; 5 Instituto de Microbiologia Paulo de Góes, Universidade Federal do Rio de Janeiro (UFRJ), Rio de Janeiro, RJ Brazil; 6 Present Address: Departamento de Microbiologia, Instituto de Ciências Biológicas, Universidade Federal de Minas Gerais, Belo Horizonte, Minas Gerais Brazil

## Abstract

Diverse pathogenic fungi secrete extracellular vesicles (EV) that contain macromolecules, including virulence factors that can modulate the host immune response. We recently demonstrated that the binding of monoclonal antibodies (mAb) modulates how *Histoplasma capsulatum* load and releases its extracellular vesicles (EV). In the present paper, we addressed a concentration-dependent impact on the fungus’ EV loading and release with different mAb, as well as the pathophysiological role of these EV during the host-pathogen interaction. We found that the mAbs differentially regulate EV content in concentration-dependent and independent manners. Enzymatic assays demonstrated that laccase activity in EV from *H*. *capsulatum* opsonized with 6B7 was reduced, but urease activity was not altered. The uptake of *H*. *capsulatum* by macrophages pre-treated with EV, presented an antibody concentration-dependent phenotype. The intracellular killing of yeast cells was potently inhibited in macrophages pre-treated with EV from 7B6 (non-protective) mAb-opsonized *H*. *capsulatum* and this inhibition was associated with a decrease in the reactive-oxygen species generated by these macrophages. In summary, our findings show that opsonization quantitatively and qualitatively modifies *H*. *capsulatum* EV load and secretion leading to distinct effects on the host’s immune effector mechanisms, supporting the hypothesis that EV sorting and secretion are dynamic mechanisms for a fine-tuned response by fungal cells.

## Introduction

Histoplasmosis is a systemic mycosis caused by *Histoplasma capsulatum*, a thermodimorphic fungus that primarily exists in a filamentous form at temperatures between 25 and 28 °C and undergoes morphogenesis to a yeast at 37 °C. Infection occurs after inhalation of micropropagules, which leads to a primary pulmonary disorder. Although infection is common, the majority of individuals have only mild or moderate symptoms. However, the fungus can disseminate throughout the body causing severe disease and death^1–3^.

The onset of the host protective response involves the rapid phagocytosis of fungal cells by neutrophils and macrophages, and the subsequent activation of a Th1 response^4,5^. Phagocytosis can occur through different molecular mechanisms, though *H*. *capsulatum* mainly engages macrophages through Fcγ receptors (IgG-opsonized *H*. *capsulatum*) and by complement receptor 3 (CR3). CR3 (CD11b/CD18) can recognize the heat shock protein 60 (Hsp60) on the surface of *H*. *capsulatum* and promote the internalization of the fungus^6,7^. Through diverse mechanisms, including regulation of vacuolar ATPases^8^ and iron acquisition^9,10^, *H*. *capsulatum* evades the host immune system and multiplies. Our group previously demonstrated that opsonization of *H*. *capsulatum* with monoclonal antibodies (mAbs) against Hsp60 can change the outcome of histoplasmosis *in vitro* and *in vivo*. In addition, administration of 6B7 mAb (IgG1) was protective while 7B6 mAb (IgG2b) failed to protect in macrophage studies or in murine infection models, despite the fact that both mAbs recognize the same epitope in Hsp60^11^. Moreover, we recently established that treatment of *H*. *capsulatum* with these mAbs significantly changed the characteristics and contents of extracellular vesicles (EV) produced by the yeast cells^12^. Fungal EV are spherical, bilayered compartments with diameters ranging from 20 to 500 nm that can carry lipids, carbohydrates, proteins, pigments and nucleic acids^13,14^, many of which are constituents of the fungal cell wall and diverse others are associated with stress response and pathogenesis, such as urease, phosphatase, catalase and laccase^15–17^. The fact that antibody directly affects EV biology extends the physiological actions of these molecules.

Given that mAb modifies *H*. *capsulatum* pathogenesis and EV content, we decided to delve deeper into associations between these findings. First, we examined if antibody-mediated changes on EV secretion and protein sorting are concentration dependent. Additionally, we assessed whether the EV produced in the presence of mAb differentially impacted host cell function by determining whether macrophages exposed to the EV had alterations in the capacity to phagocytosis and kill *H*. *capsulatum* yeast cells.

## Results

### Protein and sterol quantification in EV derived from *Histoplasma capsulatum* cells

**Figure 1:**
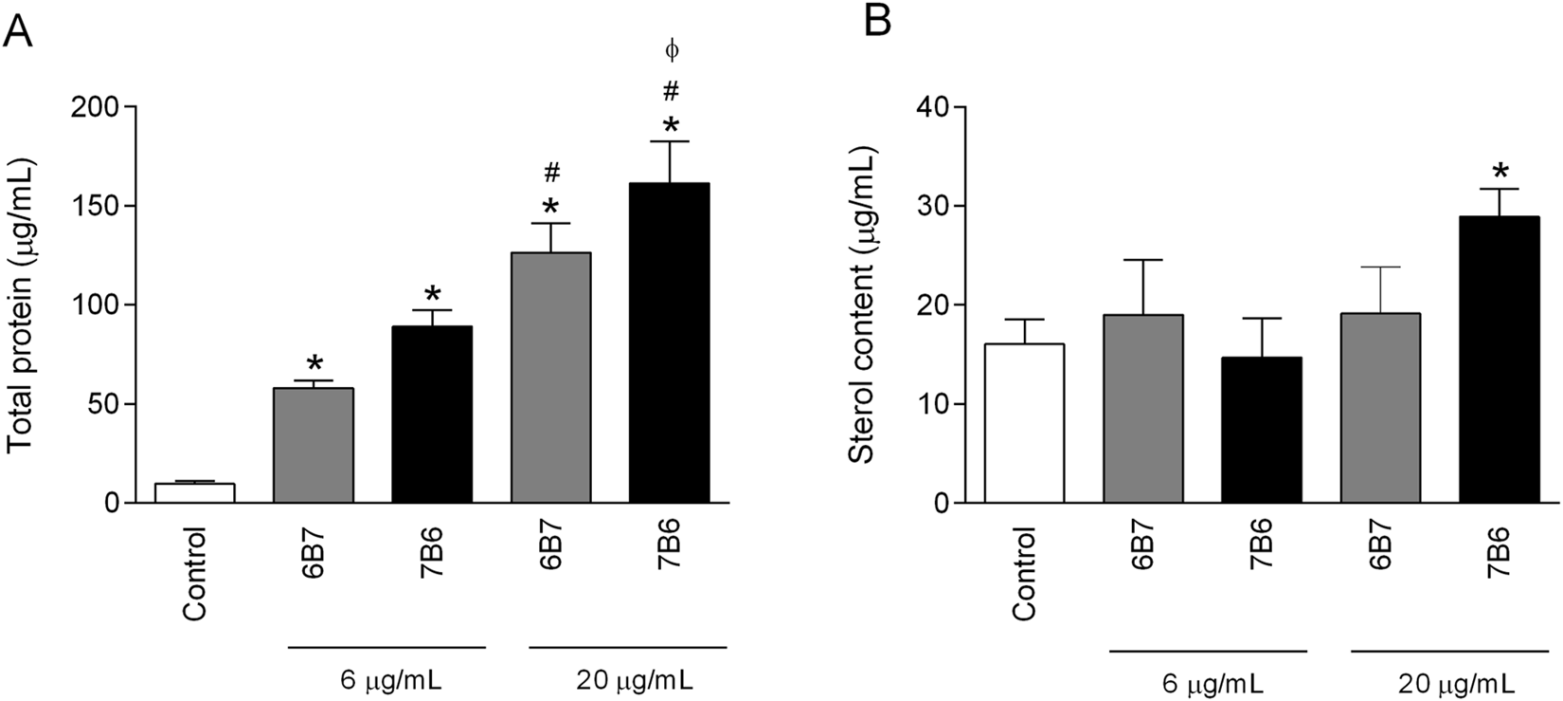
Protein and sterol quantification in EV from *H*. *capsulatum*. Yeast cells were incubated with or without 6 and 20 μg/mL of 6B7 and 7B6 mAb. The EV protein content was determined by BCA assay (**A**). EV sterol quantification was performed using Amplex reagent kit (**B**). Graphs represent means and standard deviation from at least two independent EV isolations and all the analyses were performed in duplicate. *p ≤ 0.05, compared to the untreated control group. ^#^p ≤ 0.05, compared to the groups 6B7 and 7B6 at 6 µg/mL. ^ϕ^p ≤0.05, compared to 6B7 mAb treatment at 20 µg/mL.

Protein and sterol content were evaluated in the EV’s suspension. For practical purposes, EV secreted by *H*. *capsulatum* opsonized with 6B7 and 7B6 will henceforward be referred as 6B7.6-EV and 7B6.6-EV (6 μg/mL of mAb) or 6B7.20-EV and 7B6.20-EV (20 μg/mL of mAb) respectively. Similar to what we found with 6 µg/mL of mAb^12^, 6B7.20-EV and 7B6.20-EV had an increase in the protein/sterol ratio, when compared to EV from untreated yeast cells (Fig. 1A). In addition, the 7B6.20-EV presented higher protein/sterol ratios compared to either EV from untreated yeast or 6B7.20-EV (Fig. 1B). Interestingly, an increase of sterol content was observed only in the 7B6.20-EV group. EV from *H*. *capsulatum* are divided in two populations, a smaller one with 50 nm and a bigger one with 200 nm. Both populations of 6B7.20-EV are bigger than control EV, while just the smaller population of 7B6.20-EV are bigger than the control EV (Supplementary Fig. S1).

### Enzymatic activity of fungal virulence factors in EV after treatments with mAb

**Figure 2:**
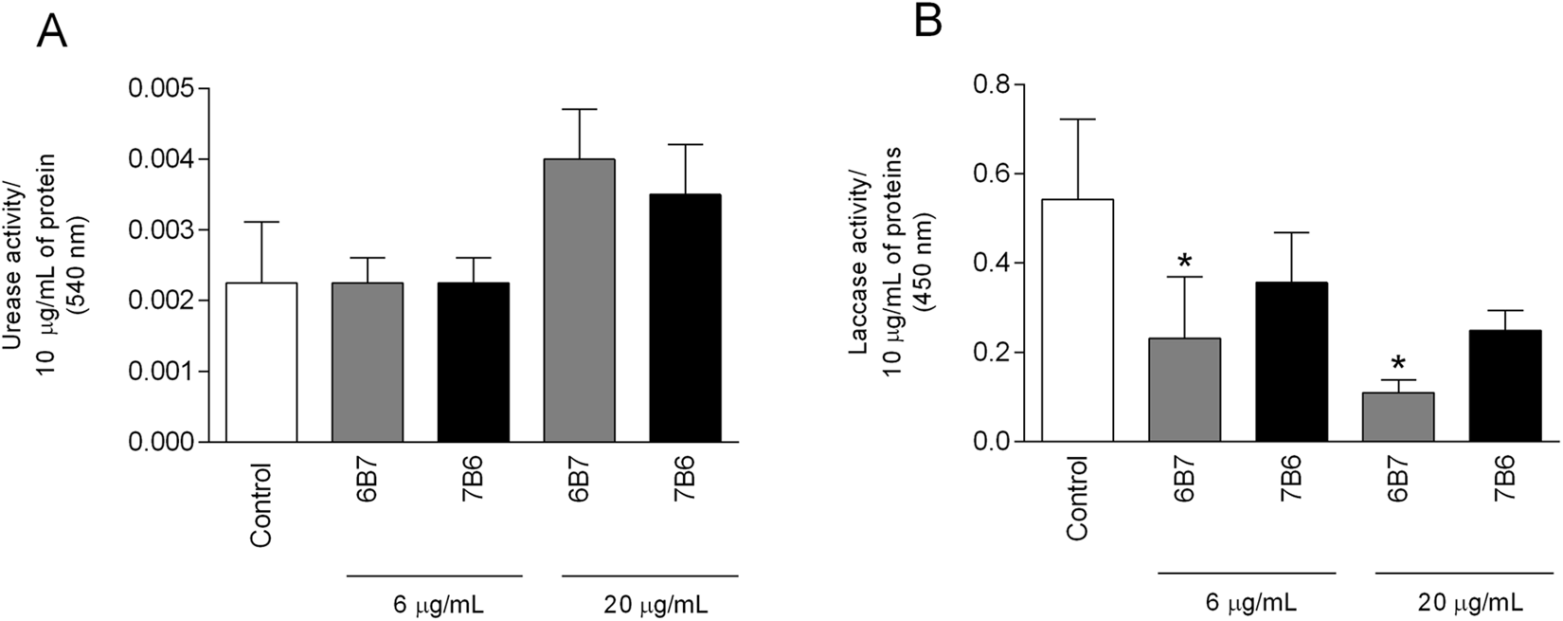
Enzymatic activity of EV virulence factors resulting from fungal opsonization with mAb. Yeast cells were incubated with or without 6 and 20 μg/mL of 6B7 or 7B6 mAb. After EV isolation, urease (**A**) and laccase (**B**) activities were measured as described in *Methods*. Graphs represent means and standard deviation of at least two independent experiments and all the analyses were performed in duplicate. *p ≤ 0.05 compared to control EV.

The activities of urease and laccase were evaluated in the suspensions of EV by the addition of chromogenic substrates specific to each enzyme. There was no difference in urease activity among the groups, even when the antibody concentration increased from 6 µg/mL to 20 µg/mL (Fig. 2A). In contrast, laccase activity was significantly reduced in 6B6.6-EV and 6B6.20-EV, but unaltered in 7B6.6-EV and 7B6.20-EV (Fig. 2B).

### Proteomic analysis of EV from *H*. *capsulatum* treated with either 6B7 or 7B6 mAb

*H*. *capsulatum* yeasts were treated with 20 µg/mL of mAbs, 6B7 or 7B6, and released vesicles were purified and analyzed by proteomic analysis. We further compared this data with our previously published data on EV derived from yeasts treated with 6 µg/mL of 6B7 and 7B6 mAbs^12^. Combining the 6 and 20 µg/mL conditions, a total of 936 proteins were detected above the limit of quantifications and use for comparative analysis (Supplemental Table S1). Combining both antibody concentration treatments, 170 and 131 proteins were differentially abundant in response to 7B6 and 6B7 mAbs, respectively, with 250 proteins differentially represented in EV resulting from *H*. *capsulatum* cultures previously incubated with mAbs (Table 1). To better understand possible functions of the EV proteins affected by the antibody treatment, we performed an annotation based on the Kyoto Encyclopedia of Genes and Genome (KEGG), followed by a function-enrichment analysis. The most affected pathways were biosynthesis of amino acids, glycolysis/ gluconeogenesis, fatty acid degradation, and vitamin B6 metabolism (Table 1). Pathways, such as biosynthesis of amino acids, ribosomes, spliceosome, protein processing in the endoplasmic reticulum might have a direct effect on the protein composition of the yeast and consequently in the EV cargo.

**Table 1:** KEGG pathway enrichment analysis of proteins with altered abundance in the extracellular vesicles after treatment of *H*. *capsulatum*. The table shows the number of proteins in each treatment on significantly-enriched pathways (*p* ≤ 0.05 by Fisher’s exact test and 2 folds enriched compared to the genome background). The analysis only considered proteins with significantly-altered abundance after treatment of *H*. *capsulatum* with 6B7 and 7B6 mAbs in the concentration of 6 and 20 µg/mL.

Pathway	Number of Significant proteins		6B7/Control				7B6/Control			
			6 µg/uL		20 µg/uL		6 µg/uL		20 µg/uL	
			Down	Up	Down	Up	Down	Up	Down	Up
			80	30	5	22	24	73	41	55
	Fold enrichment	Fisher test								
2-Oxocarboxylic acid metabolism	4.5	9.9E-03	2					2		
Alanine, aspartate and glutamate metabolism	3.8	3.6E-02	1	1				1		
Amino sugar and nucleotide sugar metabolism	4.0	1.4E-02	1			1	2		2	1
Arginine and proline metabolism	4.8	2.0E-02	1				1	1	1	1
Arginine biosynthesis	6.1	1.1E-02	1	1				1		
Biosynthesis of amino acids	7.0	4.8E-12	10	2		3	2	5	2	4
Biosynthesis of antibiotics	4.8	8.4E-12	13	3		3	5	7	4	4
Biosynthesis of secondary metabolites	4.0	2.1E-11	14	3		4	5	9	5	5
Carbon metabolism	6.1	5.2E-09	9	1		3	3	4	3	3
Citrate cycle (TCA cycle)	5.5	5.0E-03	4							
Cysteine and methionine metabolism	7.6	7.1E-06	5							3
Fatty acid degradation	10.8	6.3E-05	2				1	2	2	2
Fructose and mannose metabolism	5.8	1.2E-02	3			1	2			
Glutathione metabolism	4.8	2.0E-02	2							1
Glycolysis / Gluconeogenesis	11.5	6.5E-09	8			2	4	1	3	2
Glyoxylate and dicarboxylate metabolism	8.2	6.3E-05	4					1	1	
Lysine biosynthesis	10.1	2.6E-03	2	1						
Lysine degradation	7.0	2.0E-03	2	1			1		2	
MAPK signaling pathway - yeast	2.6	4.7E-02	3				1	1	2	
Metabolic pathways	3.2	6.8E-18	25	9		6	9	18	10	12
Methane metabolism	8.8	1.9E-04	3				1	2	1	1
mRNA surveillance pathway	5.1	8.5E-04	2	1		1		2		1
Nitrogen metabolism	7.4	6.5E-03	1	1						1
Oxidative phosphorylation	3.7	5.2E-04	3	3			1	3	2	
Pentose and glucuronate interconversions	6.1	3.7E-02	2				1	1	1	
Pentose phosphate pathway	6.7	2.4E-03	2	1		1	1	1		1
Phagosome	7.4	3.2E-05	3	1				2		2
Proteasome	3.4	4.8E-02	1	1		1		1		
Protein processing in endoplasmic reticulum	3.4	3.2E-03	7				2		3	
Pyruvate metabolism	6.6	7.5E-04	4				1		2	
Riboflavin metabolism	5.7	4.2E-02				1	1		1	1
Ribosome	4.1	5.3E-05	5	1				1	4	2
Spliceosome	3.0	6.8E-03	2	2		1				2
Tryptophan metabolism	4.4	2.5E-02	2				1		2	
Tyrosine metabolism	7.4	1.2E-04	3			1	1	1	1	2
Ubiquinone and other terpenoid-quinone biosynthesis	7.4	2.7E-02	1			1				1
Valine, leucine and isoleucine biosynthesis	5.3	4.8E-02						2		
Valine, leucine and isoleucine degradation	3.8	3.6E-02	1				1	1	2	
Vitamin B6 metabolism	10.5	1.3E-02		2						

**Figure 3:**
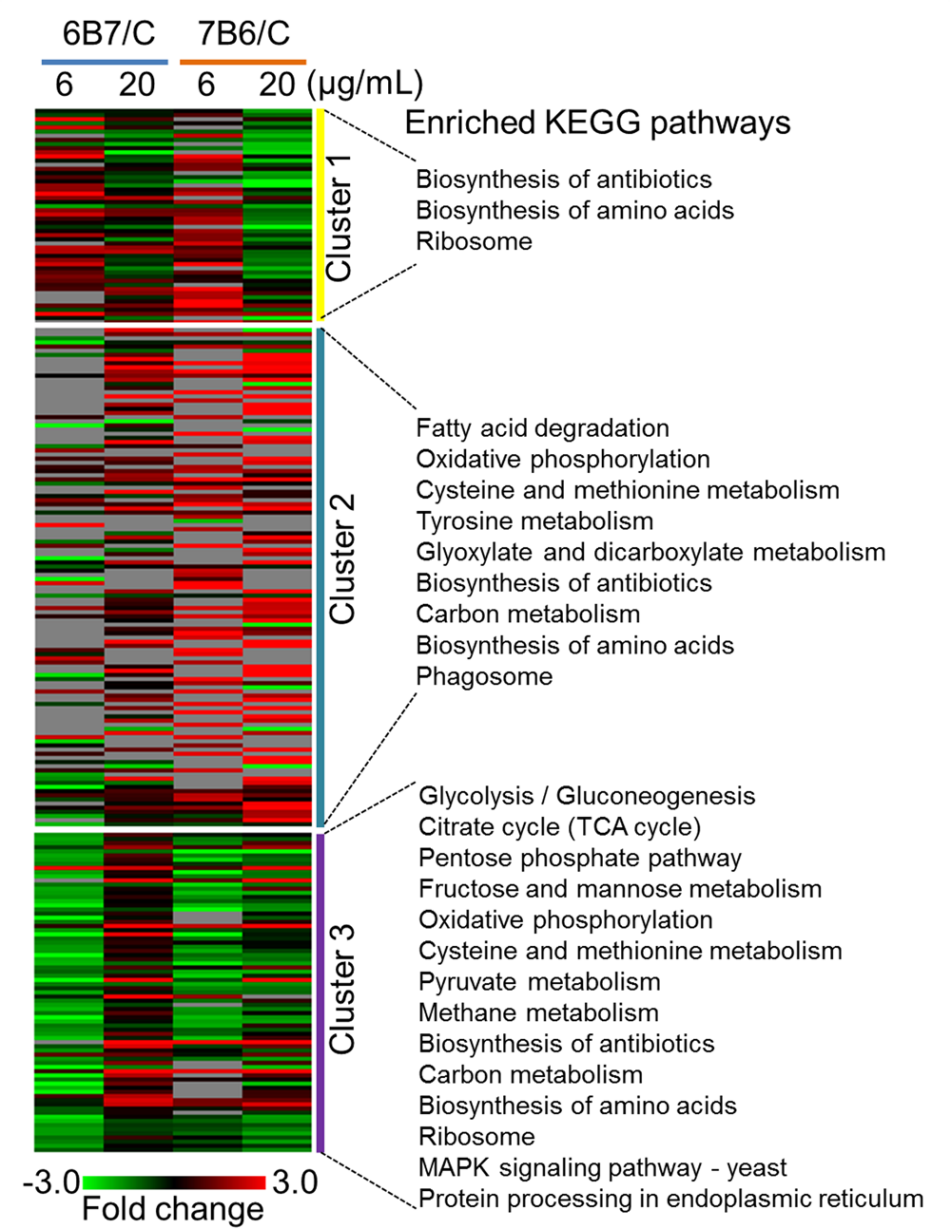
Heatmap of proteins differentially abundant in response to treatment with 6 or 20 µg/mL of 6B7 and 7B6 mAb against Hsp60. The heatmap was generated using Multiexperiment Viewer and clustered by K-means based on the abundance profiles. The significantly enriched functions (*p* ≤ 0.05 by Fisher’s exact test, 2 folds enrichment compared to the genome background) are listed for each cluster. Gray spots represent hits below the limit of quantification.

To have a view of the general trends of the antibody-induced changes in the EV composition, we clustered the proteins based on their abundance profiles using the K-mean method and plotted heat maps of each cluster. Three very distinct clusters were obtained. The first cluster had proteins whose abundances were changing in response to the antibody concentration, increased in abundances with 6 µg/mL of antibody treatment, but decreased in the higher concentration. Pathways, such as ribosomes and biosynthesis of amino acids, were enriched in this cluster (Fig. 3). The second cluster was enriched in proteins that were upregulated with the treatment, independent of the antibody concentration, and was represented by functions, such as fatty acid degradation, oxidative phosphorylation and phagosome (Fig. 3). The last cluster had proteins that were downregulated with 6 µg/mL of antibody treatment, but were less affected with the higher concentration. This cluster was particularly enriched with proteins of the central carbon metabolism (Fig. 3).

**Figure 4:**
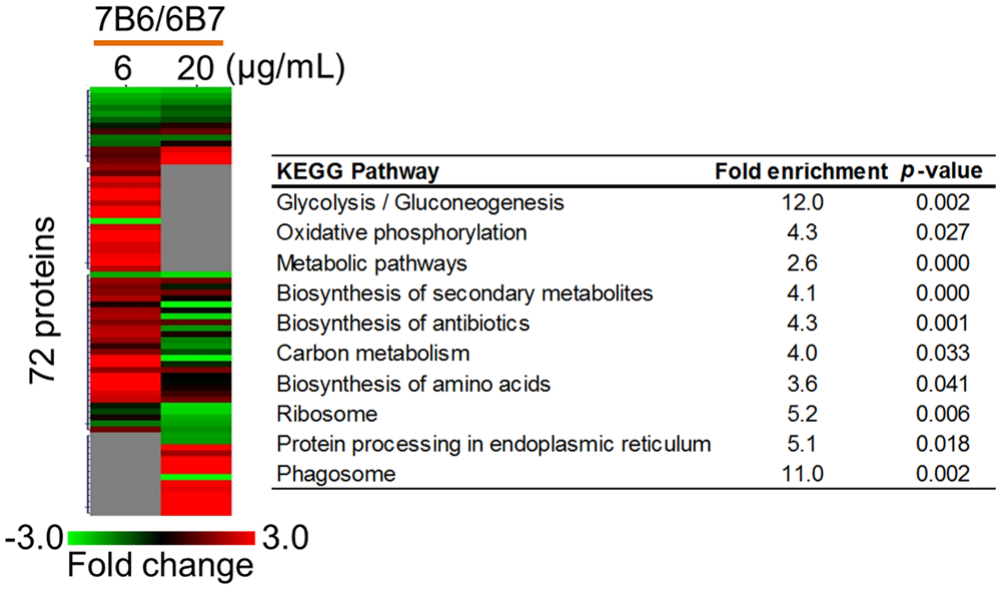
Heatmap of proteins differentially abundant in EV in response to treatment of *H*. *capsulatum* with 6B7 and 7B6 mAb against Hsp60. The heatmap was generated using Multiexperiment Viewer and clustered by hierarchical clustering based on the abundance profiles. The significantly enriched functions (*p* ≤ 0.05 by Fisher’s exact test, 2 folds enrichment compared to the genome background) are listed for each cluster. The gray spot represents hits below the limit of quantification.

We next investigated the differences in EV composition after 6B7 and 7B6 mAb treatments. A total of 72 proteins were differentially abundant comparing both antibody treatments and were overrepresented in pathways such as central carbon metabolism, protein synthesis (Ribosome and Protein processing in endoplasmic reticulum) and the phagosome. The regulation of some proteins was dependent on the antibody concentration while others were independent (Fig. 4).

### Effect of extracellular vesicles on macrophages

**Figure 5:**
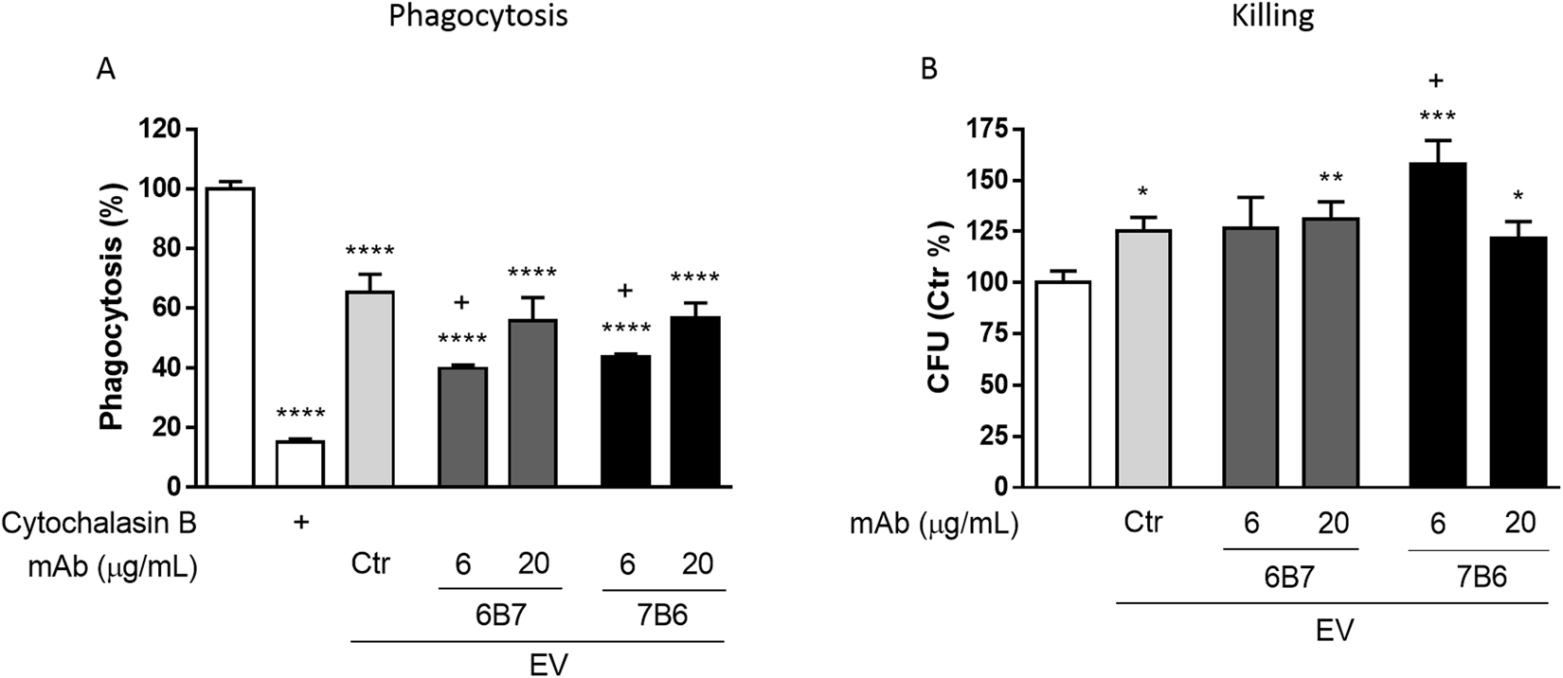
Phagocytosis and killing of *H*. *capsulatum* by EV-treated macrophages. BMDMs were treated for 1 hour with EV from *H*. *capsulatum* opsonized or not with 6 or 20 μg/mL of 6B7 or 7B6 mAb prior to the *in vitro* challenge with *H*. *capsulatum*-GFP (m.o.i. 1:5). After 1 hour, phagocytosis was analyzed by flow cytometry (**A**). To evaluate intracellular killing, after the phagocytosis time, and removal of extracellular yeast cells, macrophages were incubated for additional 2 hours and then lysed. Lysates were plated onto BHI-agar plates and colonies were counted. Graphs show the mean and standard errors from 3 independent experiments performed in triplicates. *p < 0.05; **p < 0.01; ***p < 0.001; ****p < 0.0001 compared to untreated cells, and ^+^*p* < 0.05 compared to control EV, by Student’s t-test; Cytochalasin B (10 μM).

We investigated a possible effect of distinct EV on the effector functions of bone marrow-derived macrophages (BMDM). BMDM were treated for 1 hour with EV from control or opsonized *H*. *capsulatum* prior to the *in vitro* challenge with *H*. *capsulatum* yeast cells (Fig. 5A). The inhibition of actin microfilaments dynamics with cytochalasin B largely abrogated phagocytosis. Treatment of murine BMDM with control EV inhibited *H*. *capsulatum* phagocytosis by 35% compared to non-treated BMDM. The same effect was observed when BMDM were treated with 6B7.20-EV or 7B6.20-EV. Interestingly 6B7.6-EV and 7B6.6-EV promoted an even stronger inhibition of phagocytosis compared to control EV-treated BMDM.

This data suggest that EV released by *H*. *capsulatum* can inhibit the phagocytosis of *H*. *capsulatum* by BMDM, and EV secreted by *H*. *capsulatum* opsonized with the lower concentration either 6B7 or 7B6 mAb are even more potent than control EV in inhibiting the phagocytosis of *H*. *capsulatum* yeast cells.

The intracellular killing of *H*. *capsulatum* by BMDM treated with control EV, 6B7.20-EV or 7B6.20-EV was inhibited when compared to untreated BMDM (Fig. 5B). Interestingly, the treatment with 6B7.6-EV did not promote any significant effect on the intracellular killing of *H*. *capsulatum* by BMDM, even though there was a notable trend towards the inhibition. Nevertheless, the treatment with 7B6.6-EV led to an inhibition of the yeast intracellular killing by BMDM even stronger than the control EV. These data suggest that the opsonization of *H*. *capsulatum* with the non-protective mAb (7B6) induces the release of EV that promote a more potent inhibition on the intracellular killing of the yeast by BMDM than the control EV.

The same assays have slightly different results when performed with human macrophages (THP-1-derived macrophages). The only treatment that inhibited phagocytosis was 7B6.20-EV, but the intracellular killing was inhibited by control EV as well as 6B7.20-EV and 7B6.20-EV. As seen for murine macrophages, the intracellular killing of *H*. *capsulatum* yeast cells by human macrophages was strongly inhibited after treatment with 7B6.6-EV, supporting the non-protective effect of this mAb (Supplementary Figure S2).

**Figure 6:**
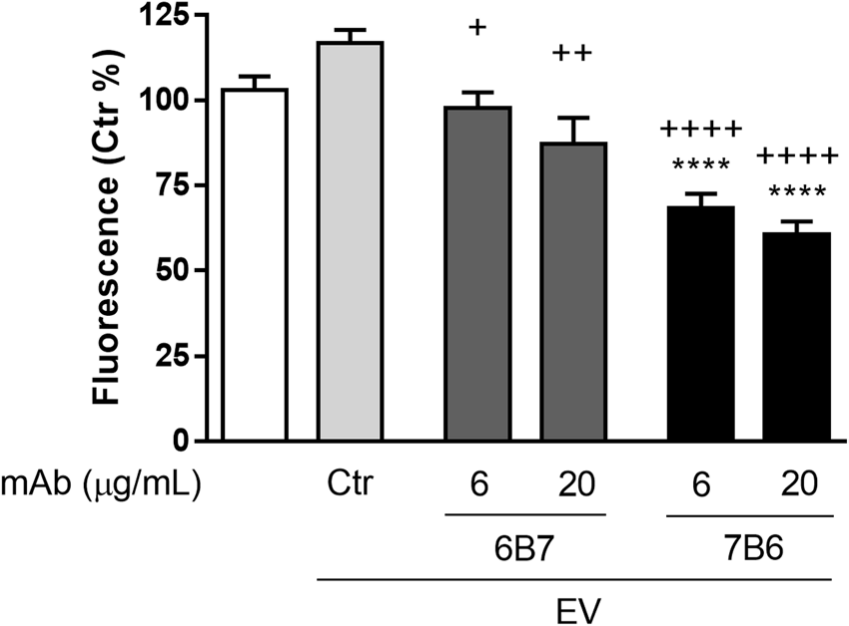
Reactive oxygen species generation by EV-treated macrophages. BMDMs were loaded with H_2_DCFDA and then treated or not with EV from *H*. *capsulatum* opsonized or not with 6 or 20 μg/mL of 6B7 or 7B6 mAb, prior to the incubation with *H*. *capsulatum* yeast cells. ROS generation was measured in a microplate reader 2 hours after the incubation of the yeast cells. Graphs show the mean and standard errors from 2 independent experiments performed in quadruples. ****p < 0.0001 compared to macrophages without EV treatment. ^+^*p* < 0.05; ^++^*p* < 0.01 and ^++++^*p* < 0.0001 compared to macrophages treated with control EV, by One-way ANOVA followed by Tukey’s multiple comparisons test.

To further address the intracellular killing inhibition of *H*. *capsulatum* by macrophages, we evaluated the reactive-oxygen species (ROS) production by macrophages that were treated with EV and then challenged with *H*. *capsulatum* yeast cells (Fig. 6).

Curiously, the treatment of BMDM with control EV promoted a (non-significant) trend in increasing the ROS generation when compared to untreated cells. However, the treatment with either 7B6.6-EV or 7B6.20-EV markedly inhibited ROS production by BMDM when compared to both untreated and control EV-treated BMDM. 6B7.6-EV and 6B7.20-EV did not promote any change in ROS generation by BMDM when compared to untreated cells, but a slight difference was noted compared to control EV-treated BMDM.

## Discussion

EV are secreted by cells from a remarkably evolutionary divergent range of organisms, including bacteria, protozoan, fungi and mammalian cells^13^. EV secretion by pathogenic organisms facilitates their survival and promotes disease through the delivery of diverse simple and complex molecules (toxins, enzymes, etc.) that subvert different hosts’ immune systems^18,19^. Given that EV from pathogenic organisms are loaded with immunogens and inflammatory activators that can efficiently trigger immune responses, EV have even been harnessed for vaccine delivery and animal models show that EV can confer protection to a host^18,20–22^. Despite advances in our knowledge of EV biology by diverse microbes, the mechanisms involved with EV biogenesis and cargo sorting as well as the roles of EV in fungal pathogenesis are poorly understood. By using mAbs against Hsp60, a protein exposed at the surface of *H*. *capsulatum* yeast cells, we recently described that mAbs have the capacity to alter the characteristics and cargo of EV^12^. In the current work we quantitatively examined alterations in protein abundance and determined whether host effector functions were affected by EV derived from *H*. *capsulatum* treated or not with 6 or 20 μg/mL of either protective (6B7) or non-protective (7B6) mAb. The amount of protein in EV samples increased when *H*. *capsulatum* was treated with either mAb at 6 or 20 μg/mL, although ergosterol concentration did not change even when the antibody concentration increased from 6 μg/mL to 20 μg/mL. The exception was for 7B6.20-EV compared to control EV. Fundamentally, mAb appears to function as a stress signal that leads the fungal cell to export more proteins through enhanced loading of EV.

The biological functions of 6B7 and 7B6 mAbs are dichotomous, with 6B7 mAb protecting the host against *H*. *capsulatum* infection, while the 7B6 mAb is irrelevant or even deleterious^11^. The hypothesis that mAbs act as a stressor for *H*. *capsulatum* is sustained by the assessment of the enzymatic activity of known virulence factors in isolated EV. Laccase is a well-known promoter of virulence in fungi^23^ and it is present in *H*. *capsulatum* yeast cells^24^. As we previously reported for EV collected from *H*. *capsulatum* treated with 6 μg/mL of 6B7 or 7B6 mAb^12^, laccase activity significantly decreased after treatment with 20 μg/mL of the protective 6B7 antibody, but not with 7B6 mAb. However, the modulation of enzymatic activity found in EV was not global, as urease activity, a virulence factor that facilitate fungal survival and proliferation in the host tissue, was not altered by the incubation of the fungus with mAb^25,26^.

The proteomic data shows that the most impacted pathways after treatment with both subtypes of antibodies were biosynthesis of amino acid, glycolysis/gluconeogenesis and fatty acid degradation. The amount of heat shock protein (C0NVB8) and heat-shock protein 60-like protein (C0P0B3) found in EV consistently decreased among all mAb treatments when compared to control, suggesting a possible feedback mechanism triggered by mAb binding to hsp-60. The presence of chitinase (C0NW75) in EV was also equally downregulated among all treatments. This data suggests that the yeast’s cell wall could be somehow impacted by the mAb binding, as we can also infer by analyzing the presence of the cell wall synthesis protein (C0ND43), which is increased after treatment with 6B7, but decreased after 7B6 mAb. The extracellular cell wall glucanase Crf1 (C0NSG6) was upregulated inside EV only after treatment with 6B7 mAb. This finding is very interesting, as this protein has proven to be highly immunogenic in *Candida albicans* and *Aspergillus fumigatus* and capable of eliciting a Th1 response in these models^27^.

In response to antibody treatments some proteins increase in abundance with 6 μg/mL of mAb, but decrease after treatment with 20 μg/mL. These differences suggest that the mAbs activate certain pathways in the yeast when in a lower concentration, but higher mAb concentrations dampen some of these pathways, by a possible desensitization of the receptor(s) involved, due to the presence of high amounts of ligand. In addition, after mAb treatment, proteins can increase in abundance independent of the antibody concentration or decrease in abundance with 6 μg/mL of antibody, but less affected with a higher concentration.

Phagocytosis of *H*. *capsulatum* is an important defense mechanism displayed by host phagocytes in order to control the infection^28^. However, during the co-evolution of pathogen and mammalian phagocytes^29^ as well as pathogen interactions with environmental predators^30^, *H*. *capsulatum* has developed a remarkable ability to live inside macrophages. Thus, the signaling pathways activated during the contact between macrophages and *H*. *capsulatum* are crucial for determining the intracellular fate of the pathogen. Interestingly, we observed that macrophages treated with EV were less able to kill fungal cells, mainly phagocytes treated with 7B6-EV. The treatment with 6B7 mAb decreased the amount of glutathione peroxidase (C0NI23) in EV. Glutathione peroxidase participates in the metabolism of glutathione, which is important for the detoxification of peroxides. Thus, the lower abundance of glutathione peroxidase in 6B7-EV could help the yeast cells to deal with peroxides generated by phagocytes. However, the intracellular killing of *H*. *capsulatum* by BMDM was slightly inhibited after the treatment with 6B7-EV but this inhibition was much stronger when BMDM were treated with 7B6-EV. This result suggests that, the scavenging of reactive-oxygen species (ROS) as well as a direct inhibition on macrophage’s NADPH oxidase and/or NO synthase are possible explanations to answer how EV increase the intracellular yeast survival.

In addition to its role as a chromatin protein, histone 2B is a cell surface antigen on *H*. *capsulatum* that is associated with binding to host cells and it also modulates the intracellular fate of the fungus^31,32^. The decrease in abundance of histone 2B after opsonization with 6B7 mAb may decrease the signal for fungal cell proliferation leading to a reduction of its intracellular replication^31^. These differences in the abundance profiles corroborate the idea that each mAb induces important changes in fungal cells that subsequently affect fungal-host interactions.

The abundance of coronin (C0NA44) and cofilin (C0P0B4), both actin cytoskeleton adaptor molecules, is consistently regulated by the yeast opsonization with either 6B7 or 7B6 mAb, while proteins such as actin (P53455), tubulin (C0NKB3), vacuolar sorting-associated protein (C0NT96) and Svf1 family protein (C0NFT1) are increased in abundance by 7B6 relative to 6B7 treatments. Differences in the abundance of these proteins suggest intense movement and high protein processing in the cytoplasm of the cell, as these proteins are involved with the rearrangement of cytoskeleton and vacuolar sorting-associated protein. Svf1 protein is required for yeast cell survival under conditions of oxidative stress; thus, its higher abundance in 7B6-EV compared to 6B7-EV suggests diminished sensitivity to exogenous reactive oxygen species^33^. These data provide new information that helps to explain some of the different features (6B7 protective versus 7B6 non-protective) observed *in vivo* by the administration of these mAb to *H*. *capsulatum* infected mice^11^.

Pre-incubation of BMDM with EV prior to *H*. *capsulatum* challenge reduced the effector cells’ capacity to phagocytose yeast cells and, curiously, EV from *H*. *capsulatum* treated with the lowest concentration of both mAb were even more potent in inhibiting phagocytosis than control EV. The concentration-independency of mAb-mediated effects on EV was also seen in the proteomic data, where the abundance of some proteins were affected by the lowest concentration of the mAb but not by the higher. The treatment of BMDM with EV for 5 or 24 hours prior to the *in vitro* challenge with the yeast did not change the fungal uptake (data not shown). Therefore, we hypothesize that the Hsp60 present on the surface of the EV (from treated or untreated *H*. *capsulatum*) competes with Hsp60 present at the surface of the yeast for the CR3 site on the surface of BMDM, thereby inhibiting yeast cell recognition. However, after longer incubations, fungal EV are endocytosed by the macrophages^16,34^, increasing the availability of free CR3 on BMDM’s surface for the recognition and uptake of the yeast cells.

BMDM were less able to kill internalized *H*. *capsulatum* after the treatment with control EV, as well as with 6B7.20-EV. However, macrophages treated with 7B6.6-EV were even less able to kill *H*. *capsulatum*, supporting the concept that 7B6 is a non-protective or even a deleterious mAb. The intracellular killing inhibition on BMDM treated with 7B6.6-EV correlates with a marked reduction in the ROS generation in these BMDM, but also suggests that mechanisms other than ROS production may regulate the intracellular fate of the yeast.

Altogether, our data show that *H*. *capsulatum* is dynamically able to sense opsonization, and, in response to mAb, the yeast’s EV cargo is quantitatively and qualitatively changed in an isotype and concentration dependent manner. Moreover, these changes appear to affect the biology and pathogenicity of the fungus.

## Methods

### Histoplasma capsulatum

*H*. *capsulatum* G217B was purchased from the ATCC (ATCC, Cat# 26032) and the GFP strain (G217B background) was kindly provided by George S. Deepe (University of Cincinnati College of Medicine, Cincinnati, OH). Fungal cells were cultivated in Ham’s F-12 (Gibco, Cat# 21700-075) media supplemented with glucose (18.2 g/L), glutamic acid (1 g/L), HEPES (6 g/L) and L-cysteine (8.4 mg/L) at 37°C with constant shaking at 150 rpm. Fungal cultures were assessed for viability using Janus green 0.02%, and >99% of the yeast cells used were alive.

### Cell Lines

All animal experiments were performed according to the Institute of Laboratory Animal Resources of the National Research Council guidelines and the Institutional Animal Care and Use Committee at the Albert Einstein College of Medicine approved the experimental methods. Balb/c mice (NCI, females aging 8–10 weeks) were euthanized and their femurs and tibias were excised and flushed with RPMI (Corning #10-040-CV) media to obtain cells. To obtain bone marrow-derived macrophages (BMDM), the cells were incubated with RPMI supplemented with 10% FBS and 20% of L929 (CLS Cat# 400260/p757_L-929, RRID:CVCL_0462) supernatant for 1 week, with media replacement at day 4^35^. Alternatively, the human monocyte cell line THP-1 (CLS Cat# 300356/p804_THP-1, RRID:CVCL_0006) was treated with PMA (Sigma #P1585) 3 nM for 48 hours at 37°C to differentiate the cells into macrophages. THP-1 - differentiated macrophages were used in some experiments specified in the text.

### mAb production

Under approval by the Einstein Institutional Animal Care and Use Committee, antibodies were generated in ascites. Briefly, mAbs were produced by the injection of 10^7^ hybridoma cells into Pristane (Sigma-Aldrich, Cat# P2870) primed peritoneal cavities of ex-breeder Balb/c female mice (NCI). Alternatively, mAb were raised from hybridoma cells (6B7, 7B6)^11^ that were cultivated in DMEM (Corning, Cat# 10-017-CM) supplemented with 10% FBS, 10% NCTC-109 (Gibco #21340-039), 1% nonessential amino acids (Corning, Cat# 25-025-CI) and penicillin/streptomycin (Corning, Cat# 30-001-CI). Supernatants were harvested, filtered and concentrated. The concentrations of 6B7 and 7B6 mAb in ascites and supernatants were determined by ELISA using known concentrations of IgG1 or IgG2b standards, respectively^12^.

### *H*. *capsulatum* treatment with protective (6B7) and non-protective (7B6) mAbs followed by extracellular vesicles purification

Exposure of *H*. *capsulatum* to mAb was performed according to a previously described protocol with minor modifications^12,15,36,37^. Briefly, *H*. *capsulatum* yeast cells were incubated with either 6 or 20 μg/mL of mAb 6B7 or 7B6. After 7 days of growth, the yeast cells were removed by centrifugation at 3,000 rpm for 10 minutes at 4 °C followed by filtration using a 0.45 μm pore filter^38^. EV purification from culture supernatant was achieved according to the protocol described by Rodrigues *et al*.^39^. Cell-free supernatant was concentrated in an Amicon ultrafiltration system with a 100-kDa cutoff membrane (Millipore #PBHK06210). The concentrated supernatant was further ultracentrifuged (Beckman Coulter Optima TLX) at 150.000 × *g* (52.000 rpm) using a TLA 100.3 rotor, (Beckman Coulter) for 1 hour at 4 °C. The EV were suspended in filtered PBS for proteomic analysis, and suspended in PBS with protease inhibitor cocktail (Roche) for dynamic light scattering analysis.

### Protein and sterol quantification

The protein concentrations in EV samples were determined using BCA reagent (Thermo-Fisher #23227) measured using a nanodrop (ND-1000 Spectrophotometer, Thermo Scientific, USA). Sterol quantifications were performed using an Amplex Red kit (Molecular Probes #A12216) according to the manufacturer’s instructions (Life technologies, CA, USA).

### Evaluation of enzymatic activities

EV suspensions with protein concentrations of 10 μg/mL were used to detect urease and laccase^17^. After addition of each enzyme reaction solution, the plates were stored at 37 °C and protected from light for 16 hours and then read using a spectrophotometer (BioTek, VT, USA). An enzyme reaction containing 1% peptone, 0.1% dextrose, 0.5% NaCl, 0.2% KH_2_PO_4_, 2% urea, and 0.0012% phenol red was used to evaluate urease activity, with plates read at 540 nm. To evaluate laccase, a solution with 12.5 mM of L-DOPA in PBS was utilized and the plates were read at 450 nm.

### Proteomic analysis

The proteomic analysis was carried out as previously described in details^12^. Briefly, EV samples from two independent isolations (biological duplicates) of *H*. *capsulatum* treated or not with 20 μg/mL of either 6B7 or 7B6, were digested with trypsin and analyzed in an Ekspert nanoLC 400 system (Eksigent) connected to a 5600 TripleTOF mass spectrometer (AB Sciex). Peptide identification was performed with the Paragon tool of Protein Pilot software (AB Sciex) by searching against the *H*. *capsulatum* complete proteome set from Uniprot Knowledge Base. Peptide peak areas were extracted with Skyline^40^ and differential abundance analysis was done using the mapDIA software (http://mapdia.sourceforge.net)^41^. The results were compared to our previously published data of EV derived from cells treated with 6 µg/mL antibodies^12^. Protein functions were automatically annotated using the BlastKOALA tool^42^. Heatmaps were generated using Multiexperiment Viewer (MeV)^43^ and proteins with similar abundance profiles were clustered using the K-means and hierarchical methods.

### Yeast Phagocytosis Assay

BMDM or THP-1-derived macrophages were plated in 12 well plates (5 × 10^5^ cells/well) and allowed to adhere for one hour. After this incubation, cells were washed with PBS to remove non-adherent cells and incubated overnight at 37^0^C at 5% CO_2_ for acclimatization. Macrophages were then incubated with 20 µg/mL (protein concentration) of EV 1 hour prior to the challenge with *H*. *capsulatum*-GFP (m.o.i. 1:5). After 1 hour of co-incubation, extracellular yeasts were removed by washing and then the macrophages were detached from the plate. Macrophages were analyzed by flow cytometry (FacScalibur – B&D) for fluorescence intensity and the percentage of infected cells determined. Data were normalized to the untreated (control) macrophages.

### Yeast Killing Assay

To evaluate the intracellular killing, BMDM or THP-1-derived macrophages were treated with EV (20 μg/mL of protein concentration) for 1 hour prior to the challenge with *H*. *capsulatum*-GFP. After a 1 hour co-culture, the extracellular yeasts were removed by washing and the plates were incubated for additional 2 hours at 37°C and 5% CO_2_. The phagocytes were then lysed with distilled water and aliquots were plated onto BHI-agar plates. After 10 days the number of yeast colonies were counted and expressed as a percentage relative to the untreated group.

#### ROS production

To address ROS production, BMDM were plated (10^5^ cells/well) in 96-well black plate. After the removal of non-adherent cells by washing, cells were loaded with H_2_DCFDA (10 μM). BMDM were then treated or not with EV (20 μg/mL of protein) for 30 minutes until the addition of *H*. *capsulatum* yeast cells. After a period of 2 hours, the plates were read in a SpectraMax M series.

### Statistical analyses

Statistical analyses were performed by T-student or One-way ANOVA followed by Tukey test, using GraphPad Prism software.

## Electronic supplementary material

Dataset 1

Supplemental Table 1
