# Supplementary material for: Concentration-dependent protein loading of extracellular vesicles released by Histoplasma capsulatum after antibody treatment and its modulatory action upon macrophages
Source: Sci Rep. 2018 May 23;8:8065. doi: 10.1038/s41598-018-25665-5 (PMC5966397; doi:10.1038/s41598-018-25665-5)
Supplement: Supplementary file 2 — Supplemental Table 1 [file 41598_2018_25665_MOESM2_ESM.pdf]

Concentration-dependent protein loading of extracellular vesicles released by *Histoplasma capsulatum* after antibody treatment and its modulatory action upon macrophages

Baltazar, Ludmila M.; Zamith-Miranda, Daniel; Burnet, Meagan C.; Choi, Hyungwon; Nimrichter, Leonardo; Nakayasu, Ernesto S.; Nosanchuk, Joshua D.

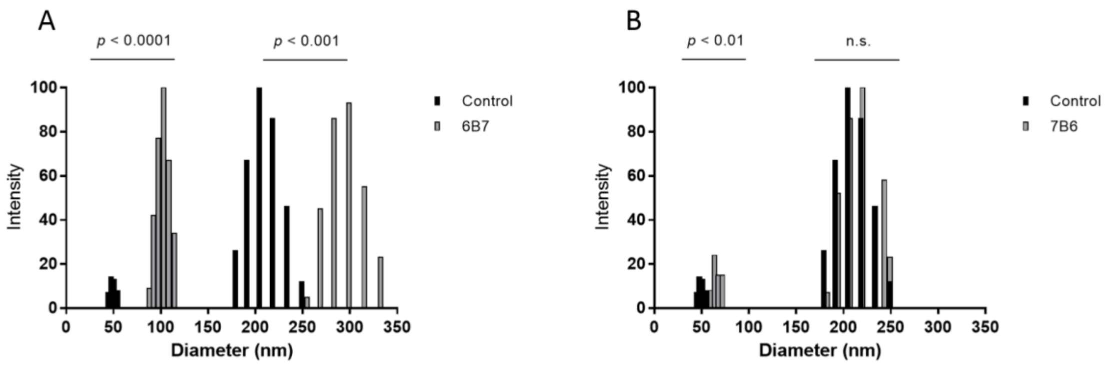

Figure S1: Effect of mAb treatment of the size distribution of EV from *H. capsulatum*. EV were isolated from cultures of *H. capsulatum* treated or not with 20 µg/mL of 6B7 (A) or 7B6 (B) mAb and subjected to dynamic light scattering analysis. Analyses were done in EV samples from 2 independent EV isolations.

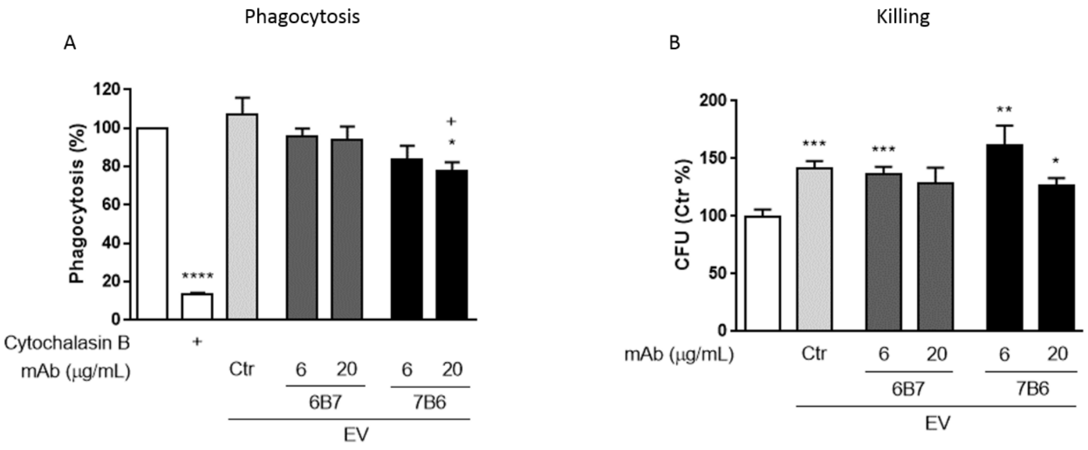

Figure S2: Phagocytosis and killing of *H. capsulatum* by EV-treated macrophages. THP-1-derived macrophages were treated for 1 hour with EV from *H. capsulatum* opsonized or not with 6 and 20 μg/mL of 6B7 or 7B6 mAb prior to the *in vitro* challenge with *H. capsulatum*-GFP (m.o.i. 1:5). After 1 hour, phagocytosis was analyzed by flow cytometry (A). To evaluate intracellular killing, after the phagocytosis and removal of extracellular yeast cells, macrophages were incubated for additional 2 hours and then lysed. Lysates were plated and colonies were counted (B). Graphs show the mean and standard errors from 3 independent experiments performed in triplicates. * = $p \leq 0.05$; ** = $p \leq 0.01$; *** = $p \leq 0.001$; **** = $p \leq 0.0001$ compared to untreated cells, and + = $p \leq 0.05$ compared to control EV, by Student's t-test; Cytochalasin B (10 μM).
